# Multi-Camera Imaging System for UAV Photogrammetry

**DOI:** 10.3390/s18082433

**Published:** 2018-07-26

**Authors:** Damian Wierzbicki

**Affiliations:** Department of Remote Sensing, Photogrammetry and Imagery Intelligence, Institute of Geodesy, Faculty of Civil Engineering and Geodesy, Military University of Technology, 01-476 Warsaw, Poland; damian.wierzbicki@wat.edu.pl; Tel.: +48-261-83-92-91

**Keywords:** UAV applications, unmanned aerial systems (UAS), photogrammetry, image matching, image mosaicking, multi-camera images

## Abstract

In the last few years, it has been possible to observe a considerable increase in the use of unmanned aerial vehicles (UAV) equipped with compact digital cameras for environment mapping. The next stage in the development of photogrammetry from low altitudes was the development of the imagery data from UAV oblique images. Imagery data was obtained from side-facing directions. As in professional photogrammetric systems, it is possible to record footprints of tree crowns and other forms of the natural environment. The use of a multi-camera system will significantly reduce one of the main UAV photogrammetry limitations (especially in the case of multirotor UAV) which is a reduction of the ground coverage area, while increasing the number of images, increasing the number of flight lines, and reducing the surface imaged during one flight. The approach proposed in this paper is based on using several head cameras to enhance the imaging geometry during one flight of UAV for mapping. As part of the research work, a multi-camera system consisting of several cameras was designed to increase the total Field of View (FOV). Thanks to this, it will be possible to increase the ground coverage area and to acquire image data effectively. The acquired images will be mosaicked in order to limit the total number of images for the mapped area. As part of the research, a set of cameras was calibrated to determine the interior orientation parameters (IOPs). Next, the method of image alignment using the feature image matching algorithms was presented. In the proposed approach, the images are combined in such a way that the final image has a joint centre of projections of component images. The experimental results showed that the proposed solution was reliable and accurate for the mapping purpose. The paper also presents the effectiveness of existing transformation models for images with a large coverage subjected to initial geometric correction due to the influence of distortion.

## 1. Introduction

Multi-camera systems, and the nadir and oblique images acquired by them, are of increasing importance in professional aerial photogrammetry. In comparison with classical photogrammetry, nadir and oblique imaging technology allow for the registration of footprints and building facades. Thanks to this, it is possible to simplify the identification and interpretation of some objects that are difficult to recognize from the unique perspective view [1,2]. Oblique images can be used to fill the existing gap between aerial images and terrestrial images [3]. Professional photogrammetry cameras are capable of mapping large areas. Multisite imaging systems Microsoft Vexcel UltraCam [4] and ZI Imaging DMC [5] generating virtual images were developed. However, these solutions are very expensive and adapted to traditional aerial photogrammetry. Recently, the use of unmanned aerial vehicles (UAVs) equipped with a compact digital camera has significantly accelerated and reduced the costs of image acquisitions [6]. UAV photogrammetry produces extremely high spatial resolution images in the short term for topography mapping [7,8], 3D modeling [9], or point cloud classification [10]. The next stage in the development of low-level photogrammetry was the development of data from UAV oblique images, where image data was acquired from side-facing directions. Similar to professional photogrammetric systems, it was possible to register footprints and facades of buildings in an urban environment. In recent years, this technique has developed significantly and become essential for the photogrammetric community. The integration of UAV photogrammetry and oblique imaging will significantly increase the range of image data obtained from low altitudes for the implementation of photogrammetry and remote sensing studies [11]. UAV equipped with a multi-camera imaging system can obtain oblique images from almost any angle of view. In the context of the development of UAV technology and multi-image matching [12], the number of studies is gradually increasing. That is why research into the implementation of the multi-camera system in UAV photogrammetry is still valid. Research in the context of the UAV is particularly important [12,13].

In addition, the use of a multi-camera system will reduce one of the main limitations of UAV photogrammetry, which is a significant lowering of the ground coverage area, associated with increasing the number of images, increasing the number of flights lines, and reducing the area imaged during one flight. One of the possible ways of increasing the range and the associated imaging area is to use several synchronized oblique and nadir cameras. At the same time, acquired images from the multiple cameras can be processed as oblique strips [14,15]. Another solution may be transformation, registration, and mosaics in order to generate one large virtual image [5,15,16].

The approach proposed in this paper is to generate a larger virtual image from five head cameras. As part of the research work, a multi-camera system was designed consisting of five GoPro action cameras to increase the total FOV without affecting the fish-eye effect. Thanks to the proposed solution, it will be possible to increase the ground coverage area and to acquire image data effectively. Virtual images will be mosaicked to limit the total number of images for the mapped area. As part of the research, cameras were calibrated to determine the interior orientation parameters (IOPs). The next section presents the method of combining images based on the scale-invariant feature transform (SIFT) and Fast Library for Approximate Nearest Neighbors (FLANN) based matcher detector and geometric correction using projective transform and the Random sample consensus (RANSAC) algorithm. The results of the mosaics of images obtained with the use of a multi-camera system are also presented.

### Related Works

Until recently, multi-camera systems were installed only on board manned aircraft. However, for several years, multi-camera systems have also been mounted on board UAV. One of the first research works on this topic was carried out by Grenzdörffer et al. [17]. As part of the research work, UAV with a multi-camera system consisting of one nadir and four oblique cameras was built. The geometric and radiometric corrections of acquired images and the possibility of applying them to automatic texture mapping of 3D-city were discussed. Other research related to UAV oblique images focused on 3D modeling of buildings [18] and trees [19]. These systems usually consist of several small and medium low-cost, compact digital cameras [15,20,21]. Thanks to such a solution, the FOV (Field of View) is significantly increased; when images from different cameras overlap, they can be mosaicked based on the geometric relationship between cameras. As a result, one virtual image can be created. By such a solution, a much larger area can be acquired in one UAV flight. Simple systems can consist of two low-cost digital cameras in vertical viewing. This solution was proposed by Tommaselli et al. [15]. In their work, they presented the next steps related to platform calibration, image rectification, and registration. Another interesting solution is a multi-camera system consisting of five digital compact cameras mounted on a large UAV AL-150 UAS platform (Aeroland UAV, Hong Kong, China). Authors of previous papers [16,22] proposed generating a virtual image based on Modified Perspective Transform. An innovative approach in the development of images from a multi-camera system was real-time bundle adjustment images from UAV as two stereo pairs [23]. Another solution was proposed previously [20]. In their work, the authors presented the concept of generating virtual images from six vertical cameras mounted on the UAV board. According to this approach, it is important to mount the cameras in a vertical position in such a way as to create an integrated structure. For each of the cameras included in the system, the interior orientation parameters (IOPs) are important, as well as for single cameras that are mounted off-nadir, the relative orientation parameters (ROPs) respective to the nadir camera [24,25,26]. Elements of the internal orientation of each of the cameras included in the system should be determined in the independent calibration procedure. In the case of relative orientation, it should be assumed that its elements are fixed for individual cameras. ROP can be determined using two methods. In the first one, elements of relative orientation are determined by differences between known elements of the external orientation (EOP) of each camera—which can be determined by observations from GNSS/IMU (Global Navigation Satellite System/Inertial Measurement Unit) sensors installed on the UAV. This method is simple, but the ROP accuracy is directly dependent on the accuracy of the measured EOP elements. That also depends on the misalignment between the GNSS/IMU onboard the UAV and the cameras. It is also based on the number and distribution of Ground Control Points (GCPs). The second method of relative orientation is based on the determination of corresponding points between the nadir camera (Master camera) and the off-nadir camera, and then on the determination of the rotation matrix and the translation vector or on the bundle adjustment. In many studies, the second method is used [15,27]. When using the second orientation method, its complexity increases with the number of cameras included in the system and their location relative to each other. The main assumption of the presented method is that the physical relations between the cameras are unchanged during the flight. In order to increase the accuracy of the designated ROPs in low-cost multi-camera systems, it is necessary to ensure adequate mutual coverage between images from individual cameras. Thanks to such coverage, the appropriate number of tie points will allow setting unknown ROPs using the bundle adjustment. Calibration and generation of virtual images with the multi-camera system mounted on the UAV can also be difficult due to the low stability of this type of platform. Wind gusts or heavy load of UAV and a relatively low flight altitude can effectively cause the whole platform to vibrate [28,29]. Therefore, one of the solutions to this problem is the use of the UAV platform presented in this article along with a dedicated 2-axis stabilized head. This solution will compensate relative movements between the cameras and will mechanically improve the stability of the cameras. This technique can be considered a bridge between classic and terrestrial image acquisition [3], and their usage in civil applications has been increasingly documented [30]. In some situations, such as the inspection of power lines [31,32,33], flexible data collection functions and high-resolution images are required, and aircraft platforms cannot meet their needs.

The contents of the paper are organized as follows. Section 1 gives the introduction and a review of related works. In Section 2, the methodology is presented. A new approach was proposed in the initial geometric correction of images obtained from the multi-camera system, and the method of the matching technique for fish-eye images is presented. Section 3 presents the research. Section 4 presents the results of research from individual stages of image processing. In Section 5, the accuracy of the proposed geometric adjustment and matching method was evaluated. Finally, Section 6 discusses the results in the context of experiments carried out by other researchers. Section 7 contains conclusions from the research and plans for further scientific research.

## 2. Methodology

The following section describes UAV platforms and a set of cameras with fish-eye lenses that were used to obtain a sequence of images. The following subchapters also present the methodology of the subsequent stages of image processing in order to obtain the mosaicked images.

### 2.1. Description of UAV Multi-Camera Imaging System

**Figure 1:**
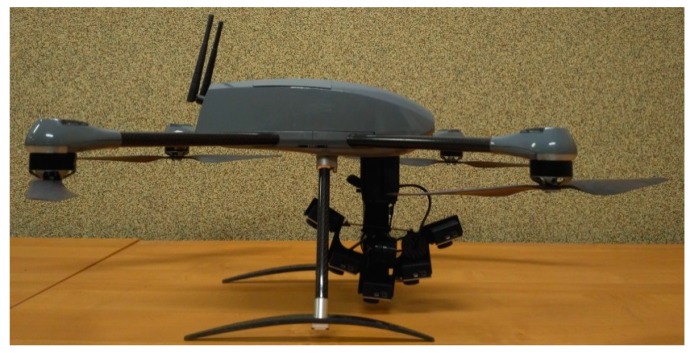
The multirotor platform adapted to carry the multi-camera system.

This chapter presents the description of the multi-camera UAV imaging system. The image data from low flying heights was obtained using the Novelty Ogar mk II platform (NoveltyRPAS, Gliwice, Poland), which can be classified to the mini multirotor category (see Figure 1).

The UAV Ogar mk II can perform air missions in beyond-visual-line-of-sight (BVLOS). The maximum takeoff weight (MTOW) of this UAV platform is 4.5 kg. Its flight time (endurance) is 40 min. The maximum speed of the UAV platform is up to 20 m/s. The system may be operated at wind speeds of up to 14 m/s and in weather conditions no worse than light intensity precipitation. UAV Ogar mk II can acquire depictions for mapping purposes in two modes—nadir and oblique imaging. Imaging in the nadir and oblique mode allows the acquisition of images to develop orthophoto maps. The multirotor ensures a completely autonomous flight at a given altitude and the given transverse and longitudinal coverage—among others thanks to the mounted GNSS/IMU receiver. The system equipment includes a flight controller that allows real-time flight management. Ogar mk II can automatically control take-off, flight, and landing. The multirotor is equipped with a stabilized gimbal. The sequences of video images are acquired in a continuous mode. For the GoPro camera set, the BLh position and Yaw, Pitch Roll angle values for the head are recorded. Flight safety is controlled automatically, but operator intervention is possible by controlling emergency safety procedures.

#### Camera Specifications

**Figure 2:**
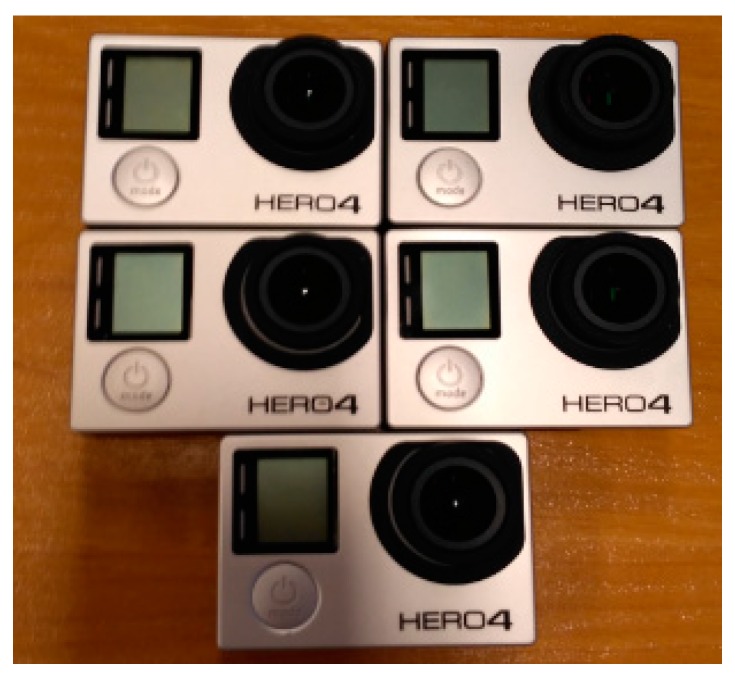
Five cameras GoPro 4 Hero Black.

In the research carried out five GoPro Hero 4 Black cameras (GoPro Inc., San Mateo, CA, USA) were used (see Figure 2) equipped with a wide-angle lens and rolling shutter. The complementary metal–oxide–semiconductor (CMOS) sensor reads images by rows. GoPro 4 camera can work in camera and video modes.

In this system the use of the different dividedness and the speeds of recording the sequence of video with different FOV (Field of View) is also possible. It records videos in 4 K/30 fps modes in ultra-wide FOV (Field of View) combination up to 170°, 2.7 K/50 fps, and Full HD/120 fps. The camera also has a fast serial mode which enables taking up to 30 pictures (12 megapixels) per second [33]. Table 1 shows the technical specification.

At present, sensors of the video are deprived of mechanical systems of the shutter for electronic rolling shutters. For camera synchronization, Smart Remote—GoPro and UAV—Mission Planner software was used.

### 2.2. Imaging Geometry for UAV Oblique Photogrammetry

For each GoPro action, the camera FOV measures the area on the surface of the Earth that is observed in a given camera by a single sensor. The area is determined based on the knowledge of the Ground Sampling Distance—GSD. The GSD for nadir was calculated using the following formula Equation (1):

(1) $$\mathrm{GSD}=\frac{p}{c_{k}}\cdot H,$$

where:

*p*—the CMOS sensor pixel size

*c_k_*—focal length derived from the camera calibration

*H*—altitude (AGL)

Table 2 shows GSD theoretical values for Nadir as a function of height and image acquisition parameters. Flight height would usually vary from 50 to 200 m for image data obtained from a low flying height. For this study a resolution of 2704 × 1520 pixels (2.7 K mode) was chosen (the central part of the image, to reduce the negative impact of image distortion caused by the camera lens) with 2.70 mm focal length.

**Figure 3:**
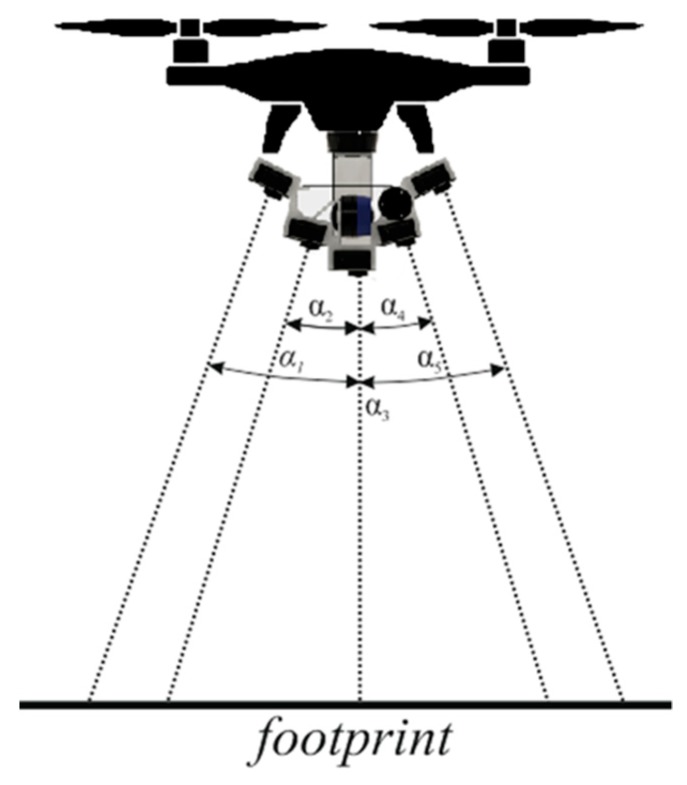
Imaging geometry for UAV multi-camera imaging system.

For oblique cameras, individual GSD values have not been determined due to the fact that, depending on the viewing angle and the time of frame capture, the scale and GSD of each image frame in different parts of the frame will be different. Figure 3 shows the acquisition geometry of the UAV oblique multi-camera photogrammetry system for 3D modeling and ortho-photomap generation. Imaging geometry is presented for roll angle.

The camera system has been designed so that a nominal overlap of the cameras is at least 70% across flight direction [11,34]. For such a system, the pitch angles of Cam1 and Cam2 cameras from the nadir are 13.2°. For Cam4 and Cam5 cameras, the pitch angles from nadir are 26.4°. The terrain range of image frames from GoPro cameras in the function of inclined from the nadir can be expressed by equations:

(2) $$\tan\alpha_{n}=\frac{footprint_{n}}{H},$$

where

*α_n_*—gimbal angle for each GoPro camera from *n* = 1 to 5

*footprint_n_*—height of photo footprint for each GoPro camera from *n* = 1 to 5

*H*—altitude

(3) $$footprint_{n}=H\cdot \left(\tan\left(\frac{HFOV}{2}+\alpha_{n}\right)+\tan\left(\frac{HFOV}{2}-\alpha_{n}\right)\right),$$

where

*α_n_*—gimbal angle for each GoPro camera from *n* = 1 to 5 [deg]

*footprint_n_*—height of photo footprint for each GoPro camera from *n* = 1 to 5

*H*—altitude

*HFOV*—vertical angle of view [deg].

### 2.3. Camera Calibration

Non-metric camera calibration allows the extraction of elements of the internal orientation for accurate 3D metric information extraction [35], those are: calibrated focal length (*c_k_*), the coordinates of the centre of projection of the image (*xp*, *yp*), the radial lens distortion coefficients (*k*_1_, *k*_2_, *k*_3_) [36], and tangential distortion coefficients (*p*_1_, *p*_2_). Therefore, it is recommended to pre-calibrate action cameras to extract reliable elements of internal orientation that allow for precise photogrammetric reconstructions. The calibration of cameras and the evaluation of the high credibility of appointed elements of the internal orientation are still an issue in the area of research of the development of photogrammetry [37] including UAV photogrammetry. Unknown internal geometry is a main problem in sensors equipped with wide-angle lenses [38,39]. The full review of camera calibration methods and models is discussed in many publications [37,40,41]. The results presented in the aforementioned articles summarize the experience associated with using digital cameras for photogrammetric measurements. It was then presented in the interpretation of different configurations, parameters, and analysis techniques of the cameras associated with the calibration. They also presented well-known photogrammetric systems from implemented models of the calibration of cameras and increasing 3D accuracy algorithms through the self-calibration bundle adjustment. The issues associated with the calibration of cameras has also become a current research topic in the field of Computer Vision (CV). Research focuses on full automatism of the process of calibration [42] on the basis of linear approaches with simplified imaging models [43]. The first work beyond these methods concerned the pinhole camera model and included the modeling radial distortion [43,44,45].

#### Camera Calibration—A Mathematical Model

Camera calibration is intended to reproduce the geometry of rays entering the camera through the projection center at the moment of exposure. The calibration parameters of the camera are:

calibrated focal length—*c_k_*;
the projection centers in relation to the pictures, determined by *x*_0_ and *y*_0_—image coordinates of the principal point;
lens distortion: radial (*k*_1_, *k*_2_, *k*_3_) and decentering (*p*_1_ and *p*_2_) lens distortion coefficients.

In the case of action cameras, there is one large FOV in wide angle viewing mode. The calibration process plays a very important role in modeling the distortion of the lens. The model of internal orientation used in the research was applied in the OpenCV based on the modified mathematical Brown Calibration model [46].

In the case of large distortion, as in the wide angle lens, radial distortion will be extended by additional distortion coefficients: 1 + *k*_4_*r*^2^ + *k*_5_*r*^4^ + *k*_6_*r*^6^. Ideally, radial distance will have the form:

(4) $$r^{2}=x^{\prime }{}_{}^{2}+y^{\prime }{}_{}^{2},$$

where:

*r*—radial distance;

*x′*, *y′*—are measured image coordinates referenced to the principal point.

When taking into account the influence of distortion, image coordinates will take the form of:

(5) $$x\prime \prime =x\prime \left(1+k_{1}r^{2}+k_{2}r^{4}+k_{3}r^{6}\right)+2p_{1}x\prime y\prime +p_{2}\left(r^{2}+2x\prime^{2}\right),$$

(6) $$y\prime \prime =y\prime \left(1+k_{1}r^{2}+k_{2}r^{4}+k_{3}r^{6}\right)+p_{1}\left(r^{2}+2y\prime^{2}\right)+2p_{2}x\prime y\prime ,$$

(7) $$\begin{array}{l} u=f_{x}\cdot x\prime \prime +c_{x}\\ v=f_{y}\cdot y\prime \prime +c_{y} \end{array},$$

where:

*k*_1_, *k*_2_, *k*_3_—polynomial coefficients of radial distortion;

*p*_1_, *p*_2_—coefficients describe the impact of tangential distortion;

*x″*, *y″*—image coordinates of point repositioning based on the distortion parameters;

*f_x_*, *f_y_*—are the focal lengths expressed in pixel units;

*c_x_*, *c_y_*—are the principal point offset in pixel units;

*u*, *v*—are the coordinates of the projection point in pixels.

In the case of camera calibration with a fish-eye lens, the calibration model in the OpenCV library is expressed using the coordinate vector of *P* in the camera reference frame is:

(8) Xc=RX+T,

where:

*R*—is a rotation matrix;

*X*—3D coordinates of *P* point

The pinhole projection coordinates of *P* is (*a*, *b*)*^T^* where: *a* = *x*/*z*, *b* = *y*/*z*, *r*^2^ = *a*^2^ + *b*^2^ and *θ* = *atan*(*r*).

The equation describing the fisheye distortion will take the form:

(9) $$\theta_{d}=\theta \left(1+k_{1}\theta^{2}+k_{2}\theta^{4}+k_{3}\theta^{6}+k_{4}\theta^{8}\right).$$

The distorted point coordinates are (*x′ y′*)*^T^* where:

(10) $$\begin{array}{l} x\prime =\left(\theta_{d}/r\right)a\\ y\prime =\left(\theta_{d}/r\right)b \end{array}.$$

Finally, conversion into pixel coordinates: The final pixel coordinates vector (*u v*)*^T^* where:

(11) $$\begin{array}{l} u=f_{x}\left(x\prime +\alpha y\prime \right)+c_{x}\\ v=f_{y}y\prime +c_{y} \end{array}.$$

At present, algorithms of the calibration of cameras were broadened by libraries open source ready answers e.g., OpenCV containing ready solutions. These algorithms are based on detecting the substantial amount of points on the flat test field of the type ‘chessboard’ [47,48]. However, the use of flat objects for camera calibration does not provide such high accuracy as 3D test fields. However, in most applications applying the 2D test fields of type ‘chessboard’ is acceptable [49,50]. For the photogrammetric purpose both mentioned methods are acceptable. The proper design of measurements, correct photography calibration tests, image measurement, and bundle adjustment allow the accurate and correct calibration for the majority of compact digital cameras.

### 2.4. Relative Orientation

The problem of the relative orientation of the cameras is to determine the 3D rotation and translation between the various cameras included in the set. Jhan [16,51] proposed that the elements of relative orientation for each camera should be calculated in such a way that the Nadir Camera (Master camera) is marked as Master, while the other Oblique cameras are marked as Slave. In this case, the angular elements of the relative orientation (ΔωPitch, ΔφYaw, ΔϰRoll) and spatial offset vectors (*V_x_, V_y_, V_z_*) for all five cameras can be determined by Equations (12) and (13):

(12) $$R_{C_{S}^{}}^{C_{M}}=R_{L}^{C_{M}}\times R_{C_{S}}^{L},$$

(13) $$r_{C_{S}^{}}^{C_{M}}=R_{L}^{C_{M}}\times \left(r_{C_{S}}^{L}-r_{C_{M}}^{L}\right),$$

where:

$R_{C_{M}}^{C_{S}}$—rotation matrix between two cameras;

$r_{C_{M}}^{C_{S}}$—the position vector between two cameras perspective centers.

**Figure 4:**
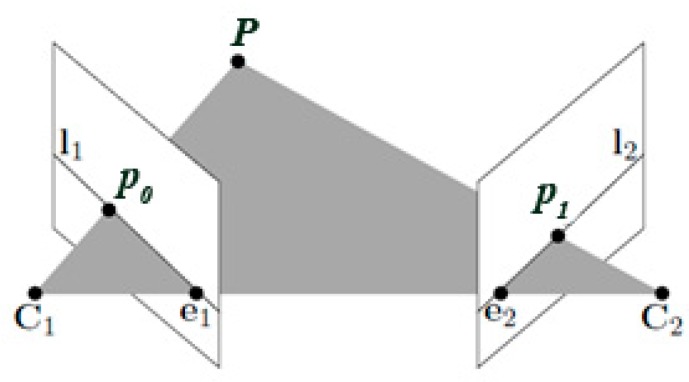
An illustration of epipolar geometry [53,54]. *P*—a 3D point; *p*_0_, *p*_1_—the projections of point P onto the image planes (normalized image coordinates); C_1_, C_2_—the baseline between the two camera centers; e_1_, e_2_—epipolar points; l_1_, l_2_—epipolar lines.

For the above equations, the relative orientation angles are calculations from $R_{C_{M}}^{C_{S}}$. It means that the rotation matrix between two cameras is a coordinated system under the local mapping frame L, where *C_M_* and *C_S_* represent Master and Slave cameras. The offset vector (*V_x_, V_y_, V_z_*) was derived from calculations of $r_{C_{M}}^{C_{S}}$ which depicts the position vector between two cameras perspective centres [16]. In the proposed approach, the elements of the relative orientation between the Master and Slave cameras were determined based on OpenCV library and epipolar geometry [52] (Figure 4).

According to theory, the main goal is to determine the rotation matrix *R* and translation vector. In the first stage of relative orientation, the search for homological points takes place using the SIFT descriptor [55] and FLANN based matcher [52]. On the basis of homological points in a pair of images, it is possible to recreate the Fundamental matrices of slave cameras. For each common point, the condition must be met [53,54]:

(14) $$p_{1}^{T}Fp_{0}=0,$$

where

(15) $$F=K_{2}^{-T}S_{t}RK_{1}^{-1}.$$

The matrix *S_t_* is the skew symmetric matrix

(16) $$S_{t}=\left[\begin{array}{ccc} 0 & -t_{3} & t_{2}\\ t_{3} & 0 & -t_{1}\\ -t_{2} & t_{1} & 0 \end{array}\right],$$

where:

*K*_1_, *K*_2_—are the calibration matrices

*R*—is the rotation of slave camera

*t*—is the translation of the slave camera

*p*_0_, *p*_1_—are images points (normalized image coordinates)

*P*—the projection point.

Then fundamental matrix *F* is determined using RANSAC and the 8-point algorithm [54,56], which defines the set of epipolar lines. The fundamental matrix is expressed in the components of the two camera matrices (relative orientation matrix—*R* and translation—*t*). The fundamental matrix has rank 2 and det(*F*) = 0 [53].

### 2.5. Rectify Action Camera Images

For geometric correction (rectification) of inclined images, the projective transformation is often used. It is an eight-parameter transformation in which information about internal and external orientation is contained. In order to determine eight coefficients of this transformation, it is necessary to know the minimum of tie points, whereby no three can lie on one straight line. For homogeneous coordinates, the projective transform can be expressed as [57]:

(17) $$\left[\begin{array}{c} x\\ y\\ 1 \end{array}\right]=\left[\begin{array}{ccc} L_{1} & L_{2} & L_{3}\\ L_{4} & L_{5} & L_{6}\\ L_{7} & L_{8} & 1 \end{array}\right]\left[\begin{array}{c} X\\ Y\\ 1 \end{array}\right],$$

where:

*x*, *y* stand for the image coordinates, *X*, *Y* for the image coordinates of reference camera (master camera), and *L*_1_, ..., *L*_8_ for the projective transformation parameters [58,59]. On this basis, the equations can be written in a linear form:

(18) $$\begin{array}{l} x=\frac{L_{1}X+L_{2}Y+L_{3}}{L_{7}X+L_{8}Y+1}\\ y=\frac{L_{4}X+L_{5}Y+L_{6}}{L_{7}X+L_{8}Y+1} \end{array}.$$

These equations are the basis for the rectification of oblique images [58,59]. A characteristic feature of projective transform is that homography transformation has eight Degrees of Freedom (DOF). The homogeneous coordinates of the adjustment points can be tied by the homogeneous matrix *H*, in such a way that for a pair of corresponding points *p* = (*x*, *y*, 1), *q* = (*u*, *v*, 1) to get:

(19) $$p\sim Hq=K_{1}R_{1}R_{2}^{T}K_{2}^{-1}q.$$

The homography matrix is a 3 × 3 matrix with an ambiguous scale. It has the following form:

(20) $$H=\left[\begin{array}{ccc} a_{11} & a_{12} & a_{13}\\ a_{21} & a_{22} & a_{23}\\ a_{31} & a_{32} & 1 \end{array}\right].$$

Because there are eight DOFs, the minimum number of points required to solve the homography is four, as shown in the following equation [60]:

(21) $$\left[\begin{array}{cccccc} x_{1} & y_{1} & 1 & 0 & 0 & 0\\ 0 & 0 & 0 & x_{1} & y_{1} & 1\\ x_{2} & y_{2} & 1 & 0 & 0 & 0\\ 0 & 0 & 0 & x_{2} & y_{2} & 1\\ x_{3} & y_{3} & 1 & 0 & 0 & 0\\ 0 & 0 & 0 & x_{3} & y_{3} & 1\\ x_{4} & y_{4} & 1 & 0 & 0 & 0\\ 0 & 0 & 0 & x_{4} & y_{4} & 1 \end{array}\right]\cdot \left[\begin{array}{c} a_{11}\\ a_{12}\\ a_{13}\\ a_{21}\\ a_{22}\\ a_{23}\\ a_{31}\\ a_{32} \end{array}\right]=\left[\begin{array}{c} x_{1}^{\prime }\\ y_{1}^{\prime }\\ x_{2}^{\prime }\\ y_{2}^{\prime }\\ x_{3}^{\prime }\\ y_{3}^{\prime }\\ x_{4}^{\prime }\\ y_{4}^{\prime } \end{array}\right]\;$$

Image coordinates can be determined according to Equations (19) and (20):

(22) $$\begin{array}{l} x_{i}^{\prime }=\frac{\left(a_{11x}+a_{12y}+a_{13}\right)}{\left(a_{31x}+a_{32y}+1\right)}\\ y_{i}^{\prime }=\frac{\left(a_{11x}+a_{12y}+a_{13}\right)}{\left(a_{31x}+a_{32y}+1\right)} \end{array}.$$

Then Random Sample Consensus (RANSAC) was used, which uses a distance tolerance to find correspondence between two sets of points to determine the transformation function. If the tolerance is too low, the process may not remove the correspondences. In the case that the tolerance value is too high, some of the correspondences may be inaccurate or incorrect. The selection of an appropriate tolerance value plays an essential role in the level of RANSAC stability and is relevant to the quality of the categorized core correspondences. The advantage of the algorithm is its simplicity and relatively high resistance to outliers even with a large number of observations. Its limitation is that with too much noise it has too many iterations, its computational complexity can be very high [61].

## 3. Research

### 3.1. Study Site and Data Set

Images obtained from low flying heights with the camera set were acquired over the test area located in the vicinity of Gliwice (Poland) (50°17′32″ N, 18°40′03″ E). The area was flat, partly wooded, and single buildings appeared on its surface. Image data from low flying heights in good weather and lighting conditions were obtained. Low grassy and shrubby vegetation covered the observed area. The test data consisted of five sets of fisheye video frames acquired with the Novelty Ogar mk II platform over the test area. A total of 100 video frames were selected for the tests. Image data was obtained from a 50 m height with GSD equal to 0.029 m in the central part of the image.

### 3.2. Proposed Approach

**Figure 5:**
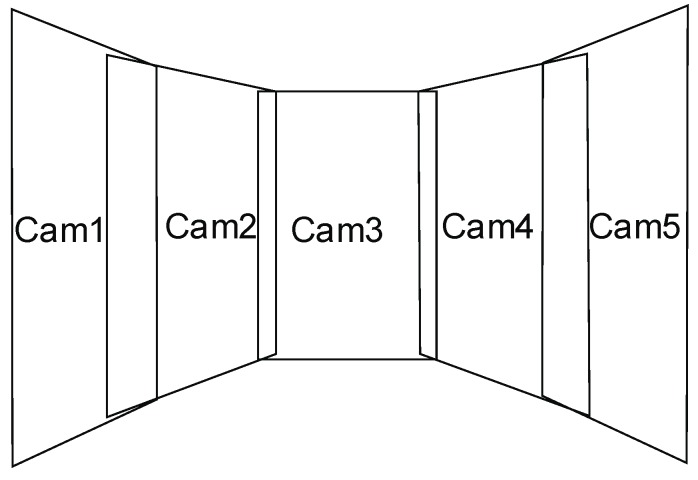
The scheme of rectification of oblique images.

The approach proposed in this article takes into account the generation of one large virtual image based on images acquired from five cameras mounted horizontally. In this configuration, the central camera is a cam-oriented camera (Cam3). However, other cameras are tilted towards central camera (Master camera) by 13.2° (Cam2 and Cam4) and 26.4° (Cam1 and Cam5), respectively (Figure 5).

Figure 5 shows a diagram of rectification of images acquired from five cameras installed on the UAV. In the proposed approach, the images are combined in such a way that the final image is a mosaic of component images.

The main stages of the proposed study are:

(a) Acquiring low-level images with cameras with the fish-eye lens;
(b) Calibration of cameras;
(c) Geometric correction of images due to distortion (Lens distortion correction);
(d) Relative orientation based on the SIFT and FLANN matcher descriptor;
(e) Projective transformation (Geometric Transform)
(f) Mosaicking to generate one large image.

**Figure 6:**
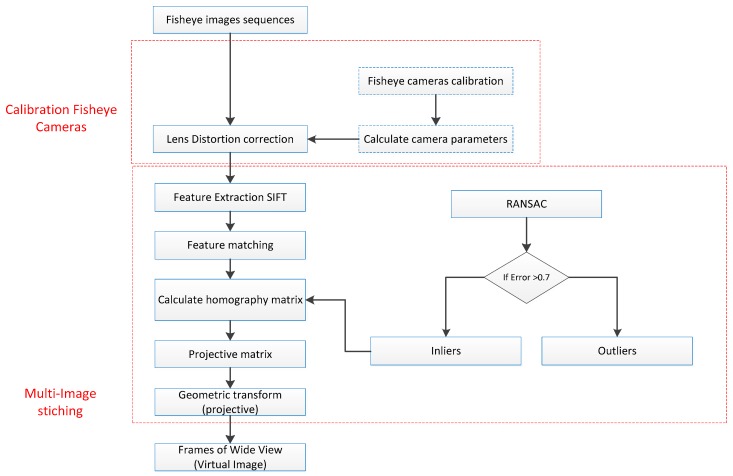
Workflow of the proposed raw image mosaicking.

The above figure (Figure 6) shows a scheme of geometric correction and mosaic of images obtained from a low flying height. First, the calibration of each camera is performed to determine the interior orientation parameters (IOPs) and distortion factors. In the further stage, the negative effect of the distortion of the lens is removed for each image. During the relative orientation of the images, tie points are found on the basis of the SIFT descriptor, and the adjustments determined using the FLANN algorithm are optimized. Next, a homography matrix is determined based on the RANSAC algorithm, for which empirically the cut-off threshold was set at 0.7. In the next stage, the Projective matrix is calculated, and the perspective transformation is performed. Then the geometric correction images are combined into a single mosaic (virtual image).

## 4. Results

### 4.1. Results of Camera Calibration

Video sequences were registered under different angles: from the front, from the right, from the left, from above, and from the ground, and all taken from the same distance. During the process of image acquisition all conditions were preserved so that the pivot of lens of each of the cameras proceeded through the focal point of the test. In this research, video modes were used to record the chessboard field at different view angles and positions. Video frames are converted to single pictures at one image per second. For each action camera five measuring series were carried out, taking into consideration in each series the accomplishment of a minimum of five frames in the different locations of the camera. During the video sequencing, similar measuring conditions were ensured for acquired samples for the most accurate results. The results of internal orientation for five cameras for the 2.7 K. Twenty calibration images for that purpose were used.

Within the framework of the research, five action cameras calibration results were in video-mode. The results obtained in both variants of calibration for the 2.7 K mode (the central part of the image mode) are comparable. The determined calibrated focal length values differ, on average, by about 0.3 mm from the given value by the producer. However, calibrated focal length and principal point coordinates are comparable with other test results [62]. The last column in Table 3 presents the results of reprojection errors for each of the calibrated cameras. The obtained error value for individual cameras ranges from 0.16 to 0.34 pixels. The most significant error value has been calculated for Cam4 camera, and it is equal to 0.34 pixels. The obtained results of the calibration of fish-eye lens cameras are comparable with the calibration results obtained by Scaramuzza et al. [63], based on the performed calibrations, the authors obtained an average reprojection error of fewer than 0.30 pixels [63].

**Figure 7:**
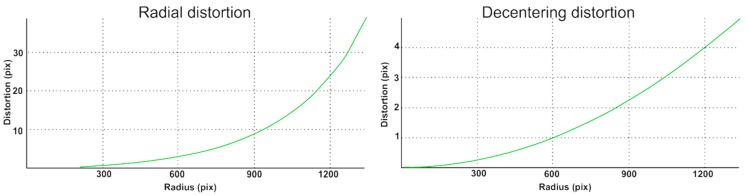
The graph with the distortion function of the cameras.

Figure 7 shows the distribution of distortion functions for an example camera that is part of the head (for other cameras, distortion functions are very similar).

### 4.2. Undistorted Fisheye Video Sequence

Based on the calibration processed on the basis of the OpenCV script, the camera matrix and distortion coefficients were developed. However, thanks to the cv2.undistort function, individual images have been rectified (the negative effect of the distortion of the lens has been removed). The undistortion method changes the position of the extreme pixels of the image and shifts them closer to the center of the image. Sometimes, some pixels are placed on the edges of the image, which distorts it. Implementation from the OpenCV library allowed the minimization of this phenomenon.

### 4.3. Visual Evaluation of the Undistortion Method

The figure below (Figure 6) shows the original image before the correction of distortion and the image after the correction of distortion.

**Figure 8:**
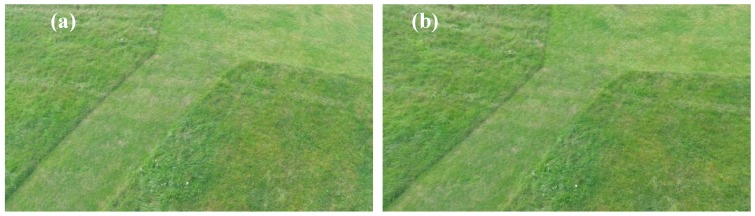
(**a**) The original image with distortion; (**b**) Undistorted image.

The proposed method of initial geometric correction noticeably (Figure 8) reduces geometric distortions in the image before the process of relative orientation. Moreover, the proposed method shows the ability to maintain angles and proportions on the stage. The advantage of the proposed method of initial geometric correction of images is the lack of distortion within the geometric image—the post-correction scene retains its original resolution.

### 4.4. Relative Orientation—Feature Image Matching

Relative orientation is performed based on generated corresponding points in every image. Corresponding points are generated based on the SIFT algorithm and FLANN matcher implemented in the OpenCV library in the Python programming environment. In SIFT algorithm, features are generated in the common area reference images. Each feature is matched by comparisons based on the Euclidean distance of their feature vectors [63]. As for matching using SIFT, the threshold of ratio test was set to 0.70.

The average number of tie points (see Table 4) generated for particular camera pairs (stereograms) ranged from 2497 to 4319. The average number of points for the camera set was 3365. The standard deviation value was 648 points. After relative orientation and image matching, the next step was to calculate the geometric transformation parameters (projective transform). In the next stage the raw matches were used to estimate the fundamental matrix. For this purpose, it was necessary to reject outliers and select inliers for the correct determination of geometric transform.

After every matrix estimation, a matrix validation test is performed to avoid a distorted transformation based on incorrect matches.

(a) Torsional factor in homography (*H*_3.1_, *H*_3.2_) cannot be too significant. Its absolute value is usually less than 0.002.
(b) A shift between images is not allowed when combining images. The homography is rejected if it changes the x and y coordinate between itself.

In an unrelated combination, it should be decided whether the two images match or not. The number of estimated matches (inliers) can be one of the criteria. However, high-resolution images often have outliers (see Table 5). A sufficient number of iterations in RANSAC should eliminate this problem. Also, it should be noted that incorrect matches are often randomly placed on the image. An additional criterion of geometry can also improve the accuracy of image matching [64,65].

**Figure 9:**
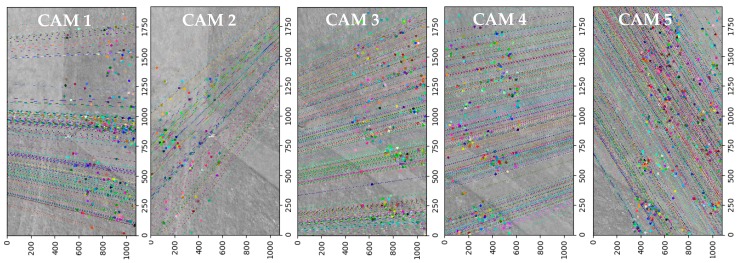
The distribution of matching points (using the best correspondences) for images acquired by every camera.

As can be seen from the Figure 9 analysis, after optimization, the density of tie points has been significantly reduced. On the basis of the RANSAC algorithm, only points (inliers) meeting the cut-off criterion were used to transform the images.

## 5. Accuracy Assessment of Rectifying Results

### 5.1. Results from Multi-Camera Matching

Table 6 presents the results of the relative matching of images after transformation. Relative orientation in the proposed approach was made to the pixel level. For each pair of images, the mean square error (RMSE) values were determined.

Based on the analysis of the obtained results, it can be seen that the highest stability was characterized by a pair of images acquired using Cam1 and Cam2. In this case, the RMSExy value was only ±2.13 pix. In the case of stereograms acquired from Cam2 and Cam3 cameras, the accuracy of image matching was almost 4 pixels, more precisely RMSExy = ±3.80 pix. The most significant error value for these stereograms was probably caused by a presence on the photographed scenes—an object being in motion (a moving person). The average value of the RMSE error for matching all images was ±3.18 pix.

### 5.2. Result of Image Stitching

**Figure 10:**
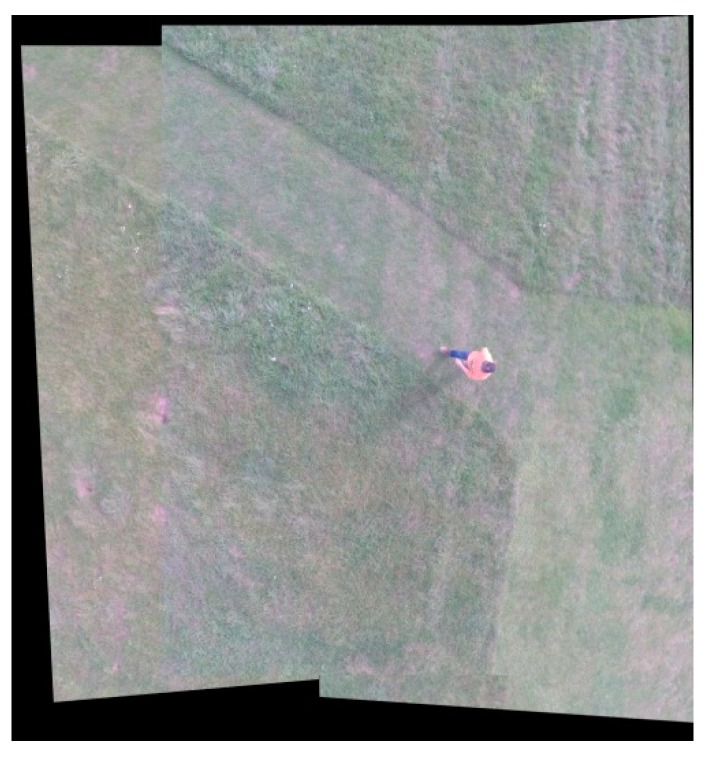
The correction results after relative orientation and geometric correction of two stereograms.

**Figure 11:**
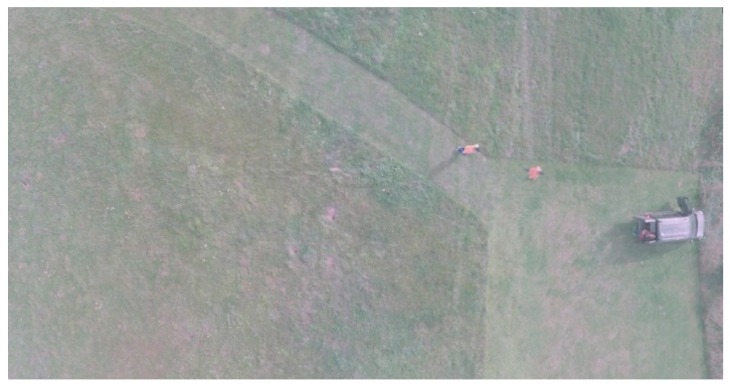
The final mosaicking result from five cameras.

Figure 10 shows the mosaics on the example of two stereograms. At the initial stage of the study, the adverse influence of distortion was removed, then the relative orientation and geometric correction of the images were made. The part of the presented mosaic is the introduction to the final form of the images presented in Figure 11. The average distance between the calculated and the actual location of the point was slightly over 3 pixels. The most substantial image distortion was recorded at the edges of the mosaic.

During the development of the mosaic consisting of a nadir image and four off-nadir images, it will still be geometrically distorted relative to the orthoimage. Therefore, the images that have been mapped must be subjected to the classical orthorectification process, taking into account the influence of the relief, which was not the subject of this study, so that they could finally be presented from the nadir view. The proposed process of developing one large image can effectively increase the coverage area. A small limitation of the proposed method on mosaic images may be the adverse phenomenon of ghosting of objects in motion.

## 6. Discussion

As part of the performed research, a method for acquiring, calibrating, geometric correction, and combining images obtained from a low-level was proposed. The presented method allows the integration of images obtained from the multi-camera system installed on the UAV board. The proposed method of registering multiple images with the help of a multi-chamber system will allow for the issuing and timely registration of remote sensing data, which can be successfully applied in environmental mapping and change detection. Increasing efficiency in obtaining oblique images from UAV was also observed during research work carried out previously [2]. The authors proposed using SIFT descriptor and feature matching to orientate oblique images. In their work, they stated that combining tiling strategy with existing workflows can provide an efficient and reliable solution. Also, in a previous paper [30], the orientation process of oblique aerial images is presented based on the Binary Robust Independent Elementary Features (BRIEF) descriptor. The effective method of geometric correction of remote sensing images using the SIFT and Affine-Scale Invariant Feature Transform (ASIFT) algorithm is also presented in a previous research [66], where the authors obtained geometric correction errors in the range from 0.63 to 3.74 pixels in the image defocus function from 30° to 70°. In other studies [67], similar results were obtained by mosaicking UAV images based on SIFT descriptor and RANSAC to remove the wrong matching points. Based on the experimental results, it was proved that the proposed solution could effectively reduce the impact of accumulative error and improve the precision of the mosaic while reducing the mosaic time by up to 60%. The accuracy of the geometric correction and multi-camera mosaic of images was also studied in a previous paper [20]. The authors used the six-camera system in their temperaments and achieved the accuracy of mosaic and geometric correction of images, which was 3 pixels. A similar result was achieved in the experiments presented in this article, where the average RMSE value was 3.18 pixels.

The main limitation of the proposed method is that it works effectively in the case of images acquired by cameras, mutual stability must characterize them—they should be placed in one frame. In addition, it is also sensitive to the tonal heterogeneity of acquired images. However, a similar allegation can be made about other systems acquiring image data obtained from a low flying height. Another limitation is the fact that there should not be objects in motion in the photographed area (as the example of the Cam2-Cam3 stereogram in which an object in motion was photographed, the accuracy of image matching was the lowest). Also, in such cases, erratic estimation of homography is possible for oblique images, which leads to the inaccurate geometric correction of images.

## 7. Conclusions

Until now, the possibility of acquiring images from the multi-camera imaging system installed on the UAV multi-copter was not taken into account in environmental mapping. The basic methods of geometric correction are insufficient to accurately correct images acquired with fisheye-lens cameras, which are additionally mounted diagonally. On the basis of the above premises, a method for the geometric correction of images and their combination into one virtual image was developed. The proposed method takes into account the correction of distortions. This approach allows the effective binding of the tie points and also improves the accuracy of the geometric transformation. The proposed method of image integration can increase the area of imaging by the UAV multirotor. Based on the above, it is evident that the multi-camera system has a higher dynamic range in relation to individual cameras equipped with normal or wide-angle lenses. The performed tests are particularly important in the context of geometric correction of remote sensing images and in environment mapping. Future research will focus on taking into account tonal differences in component images and fully automating the processing of large sets of images. In addition, it is planned to implement the proposed UAV fixed-wing application. Thanks to this it will be possible to increase the imaging area even more effectively. Additionally, in future research work, the use of geometric properties of cameras in the use of effective 3D modeling of buildings is planned.

## Figures and Tables

**Table 1:** Technical specification of GoPro 4 Hero Black camera.

Item	Description
Size [mm]	41 × 59 × 30
Weight [g]	88
Optical sensors type	CMOS
Digital Video Format	H.264
Nominal focal length [mm]	3
Image Recording Format	JPEG
Max Video Resolution	3840 × 2160
Effective Photo Resolution	12.0 MP
Sensor size [mm]	6.16 × 4.62
Pixel pitch [µm]	1.55
Sensor width [mm]	4.19
Sensor height [mm]	2.36

**Table 2:** Calculated GSD and FOV for GoPro action camera in Nadir.

Flight Height [m]	GSD [m]	HFOV Nadir [m]	VFOV Nadir [m]
50	0.029	77.61	43.63
75	0.043	116.42	65.44
100	0.057	155.23	87.26
125	0.072	194.04	109.07
150	0.086	232.84	130.89
175	0.100	271.65	152.70
200	0.115	310.46	174.52

Note: horizontal field of view (HFOV); vertical field of view (VFOV).

**Table 3:** Calibration results for five cameras for 2.7 K Video mode GoPro4 Hero Black.

Parameter	CAM 1		CAM 2		CAM 3		CAM 4		CAM 5	
	Mean Value	σ	Mean Value	σ	Mean Value	σ	Mean Value	σ	Mean Value	σ
*c_k_* [mm]	2.70	0.015	2.70	0.001	2.77	0.025	2.72	0.041	2.79	0.019
*x*_0_ [mm]	0.144	0.074	0.150	0.003	0.145	0.050	0.130	0.041	−0.297	0.008
*y*_0_ [mm]	−0.056	0.007	0.024	0.005	0.096	0.051	0.069	0.076	0.025	0.107
*k*_1_	4.56 × 10^−4^	2.31 × 10^−6^	4.59 × 10^−4^	3.72 × 10^−8^	4.62 × 10^−4^	2.26 × 10^−6^	4.61 × 10^−4^	1.36 × 10^−6^	4.56 × 10^−4^	1.42 × 10^−6^
*k*_2_	2.70 × 10^−7^	1.87 × 10^−8^	2.89 × 10^−7^	9.72 × 10^−10^	2.65 × 10^−7^	1.98 × 10^−8^	2.57 × 10^−7^	1.21 × 10^−8^	2.80 × 10^−7^	1.18 × 10^−8^
*k*_3_	3.86 × 10^−11^	3.98 × 10^−11^	1.75 × 10^−11^	2.73 × 10^−12^	3.10 × 10^−11^	4.88 × 10^−11^	1.06 × 10^−10^	2.85 × 10^−11^	3.51 × 10^−11^	2.60 × 10^−11^
*k*_4_	−3.27 × 10^−2^	2.72 × 10^−3^	−9.10 × 10^−2^	3.12 × 10^−3^	−1.99 × 10^−2^	1.09 × 10^−3^	−1.17 × 10^−2^	2.93 × 10^−3^	−8.30 × 10^−2^	1.32 × 10^−3^
*p*_1_	6.00 × 10^−5^	3.49 × 10^−5^	−3.46 × 10^−5^	1.00 × 10^−6^	9.87 × 10^−5^	3.29 × 10^−5^	2.91 × 10^−5^	2.44 × 10^−5^	5.68 × 10^−5^	1.30 × 10^−5^
*p*_2_	3.46 × 10^−6^	4.85 × 10^−6^	−6.34 × 10^−5^	2.54 × 10^−6^	−3.42 × 10^−4^	3.09 × 10^−5^	6.04 × 10^−5^	5.00 × 10^−5^	−1.85 × 10^−4^	6.49 × 10^−5^
Reprojection error [pix]	0.29	0.24	0.16	0.34	0.26	0.29	0.24	0.16	0.34	0.26

**Table 4:** The number of raw matches for stereograms.

Stereograms	Cam1 and Cam2	Cam2 and Cam3	Cam3 and Cam4	Cam4 and Cam5
Raw matches	2497	3397	3246	4319

**Table 5:** Results of matching images from individual cameras.

Cameras	Inliers (RANSAC)	Fundamental Matrix Error
Cam1 and Cam2	249	−0.002334
Cam2 and Cam3	332	−0.000219
Cam3 and Cam4	380	0.000074
Cam4 and Cam5	682	0.032159

**Table 6:** Results from multi-camera matching.

Image Pairs	RMSE_x_ [pix]	RMSE_y_ [pix]	Total RMSE_xy_ [pix]
Cam1 and Cam2	1.67	1.32	2.13
Cam2 and Cam3	2.48	2.88	3.80
Cam3 and Cam4	2.59	2.09	3.32
Cam4 and Cam5	2.58	2.28	3.45
